# Effects of tissue flossing and dynamic stretching on hamstring stiffness and flexibility in light volleyball enthusiasts: a randomized controlled trial

**DOI:** 10.3389/fphys.2025.1703100

**Published:** 2025-12-16

**Authors:** Kang Ren, Zhendong Zhao, Lu Wu, Liyu Luo

**Affiliations:** 1 School of Physical Education and Health Engineering, Taiyuan University of Technology, Taiyuan, Shanxi, China; 2 Department of Physical Education and Research, China University of Mining & Technology, Beijing, China; 3 Shanxi Road & Bridge Intelligent Transportation Information Technology Co, Ltd., Jinzhong, Shanxi, China; 4 School of Sport Medicine and Physical Therapy, Beijing Sport University, Beijing, China

**Keywords:** tissue flossing, dynamic stretching, hamstring stiffness, shear-wave elastography, light volleyball, ultrasound image, myofascial release

## Abstract

**Introduction:**

This randomized controlled trial aimed to compare the acute and sustained effects of tissue flossing (TF) versus dynamic stretching (DS) on hamstring muscle stiffness and flexibility in light volleyball (LVB) enthusiasts.

**Methods:**

Thirty-seven participants were randomly assigned to TF, DS, or placebo groups. Muscle stiffness was evaluated using shear-wave elastography, measuring Young’s modulus at the distal and proximal regions of the biceps femoris long head and semitendinosus. Flexibility was assessed via passive knee extension (KE), straight leg raise (SLR), and forward flexion distance (FFD) at baseline, immediately post-intervention, and 30 min post-intervention.

**Results:**

The results demonstrated that TF significantly reduced muscle stiffness compared to DS at the 30-min mark, particularly in the semitendinosus [distal: mean difference = −43.40 kPa, 95% CI (–80.65, −6.16), p = 0.007; proximal: −51.13 kPa, (–101.20, −1.05), p = 0.040]. However, no significant differences were observed between the TF and DS groups in flexibility outcomes (KE, SLR, or FFD) at any time point.

**Discussion:**

These findings indicate that while TF offers a prolonged reduction in hamstring stiffness—suggesting potential benefits in injury prevention and prolonged performance—its effect on functional flexibility remains comparable to that of dynamic stretching. TF may serve as an effective warm-up intervention for athletes requiring sustained decreases in muscle stiffness during activities.

## 1 Introduction

Light volleyball (LVB) is a newly adapted physical activity derived from traditional volleyball. Compared with volleyball, LVB has lower physical and competitive requirements for the participants and is a sport that combines both fun and competitiveness (Leung et al., 2025). LVB and volleyball have similar technical characteristics, but LVB participants are mostly sports enthusiasts who lack professional training, so they often suffer from similar musculoskeletal injuries as traditional volleyball players. Hamstring strain (HS) often occurs during the sudden take-off and landing stage in volleyball (Dalton et al., 2015; Erickson and Sherry, 2017). It is related to the excessively high stiffness and insufficient flexibility of the hamstring tissue (Bradley et al., 2007; Freitas et al., 2023; Witvrouw et al., 2003). Once an HS occurs, athletes often experience acute pain, swelling, and lower limb motor dysfunction. Moreover, after recovery from the injury, the flexibility of the hamstrings further declines, which not only increases the risk of re-injury but also seriously affects the athlete’s performance (Liu et al., 2012).

It is important to distinguish between muscle flexibility and passive muscle stiffness as they represent related but distinct physiological properties. Flexibility typically refers to the range of motion around a joint and is influenced by factors including muscle extensibility, neural tolerance to stretch, and joint mechanics (Takeuchi et al., 2021; Yu et al., 2022). Passive muscle stiffness, in contrast, is an intrinsic mechanical property of the muscle–tendon unit, reflecting its resistance to an external stretch force, which can be quantified by techniques such as shear-wave elastography (SWE) (Deng et al., 2023). While excessive stiffness is considered a risk factor for strain injuries, optimal levels of stiffness are also crucial for force transmission and athletic performance.

Dynamic stretching (DS), as a traditional warm-up method, is usually the preferred option for athletes to prevent strains before exercise (Cai et al., 2023; Iwata et al., 2019). DS can immediately improve athletes’ flexibility, increase the joint range of motion, and activate muscle performance to prevent the occurrence of HS (Behm et al., 2016; Hough et al., 2009). However, in games, HS occurs more often in the final period before each half or late in the overall game (Rasp et al., 2024; Yagiz et al., 2022). Recent studies have shown that the improvement effect of DS on hamstring flexibility rapidly declines within 30 min (Cai et al., 2023; Opplert and Babault, 2018). This may be because the improvement in flexibility by DS is achieved more through the activation of muscle spindles to produce acute neural adaptation, with little improvement in tissue stiffness (Takeuchi et al., 2022; Vieira et al., 2021). Therefore, maintaining the continued improvement in hamstring stiffness after warming up may be the key to preventing HS during exercise.

Tissue flossing (TF) is a new method aimed at improving athletes’ joint range of motion, reducing pain, and preventing injuries. TF usually involves wrapping a rubber elastic band around a muscle or joint, coordinating with the contraction or sliding of the tissue, and removing it within 2 min to release the fascia tissue. Although unproven, the underlying mechanism by which TF promotes myofascial gliding through compression and reduces tissue stiffness is a key hypothesis for its application to improve flexibility (Konrad et al., 2021; Maemichi et al., 2025; Maust et al., 2021). Existing research indicates that TF can immediately improve the tissue stiffness of the hamstring muscle and enhance its flexibility (Kaneda et al., 2020; Wu et al., 2022). Therefore, we believe that using TF as a warm-up method for LVB may decrease the occurrence of HS. We conducted a single-blind, randomized controlled trial to compare the immediate and continuous effects of TF versus DS on hamstring stiffness and flexibility. Specifically, we hypothesized that TF would yield advantageous immediate and sustained improvements in hamstring stiffness and flexibility in LVB enthusiasts compared to DS.

## 2 Materials and methods

### 2.1 Participants

A total of 37 healthy LVB enthusiasts (male = 23, female = 14, age = 25.65 ± 6.17) were recruited. The inclusion criteria were as follows: physical capability to perform the test movements (e.g., no dizziness during the standing forward flexion test), tolerating the intervention intensity, and no history of HS injury within the previous 3 months (Aparicio et al., 2009). The exclusion criteria were as follows: having a history of lumbar disc herniation or protrusion, symptoms of ligamentous laxity, and contraindications for TF, including latex allergy, hypertension (resting systolic blood pressure >160 mmHg or diastolic >100 mmHg), venous thromboembolic disease, cardiac disease, respiratory disease, or significant neurological, orthopedic, dermatological, or neuromuscular issues in the lower leg (Kaneda et al., 2020). Following a detailed explanation of the experimental procedures, all participants provided written informed consent. The study protocol received ethical approval from the Institutional Review Board affiliated with one of the authors (approval no. TYUT2025070402). The recruitment of participants was completed between 2 July 2025 and 15 July 2025.

### 2.2 Protocol

In this single-blind, randomized controlled trial, participants were assigned to one of three groups: the TF, DS, and placebo groups. Participants completed the measurements and interventions in the school’s constant-temperature laboratory at 26 °C. The hamstring flexibility test and tissue stiffness test of different parts of the hamstrings were measured at three time points: before the intervention (baseline) and 0 and 30 min after the intervention.

**FIGURE 1:**
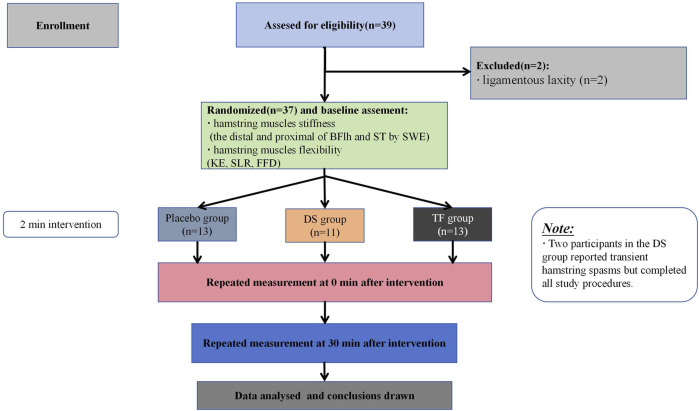
Study flowchart.

The participants were instructed to maintain their normal dietary habits and refrain from vigorous physical activity for 2 days before the experiment. During the whole experimental period, all participants were required not to perform flexibility training or any intervention that affects flexibility (such as static stretching or massage) other than the experimental group requirements. These requirements were detailed in the informed consent form; participants who did not comply with them were withdrawn from the intervention, and their data were excluded. The trial began on 16 July 2025 and ended on 31 July 2025. A flowchart of the study procedures is shown in Figure 1.

### 2.3 Randomization, allocation concealment, and blinding

Participants were randomized into the TF, DS, or placebo groups. Randomization was completed using stratified permuted block randomization by a research assistant who was not involved in participant recruitment. Blocks were completed in groups of nine assignments each. Index cards with concealed group allocation were folded and sealed in envelopes. The envelopes were shuffled by three independent researchers and placed in folders corresponding to each group by the research assistant. This was completed for each group. The process was repeated once the envelopes were used for each group. After the initial assessment was completed and consent was received, the blinded assessor informed the therapist of the corresponding group. Outcome assessors remained fully blinded throughout all evaluations, with no access to the intervention records.

### 2.4 Interventions

All participants received a 2-min warm-up intervention targeting the dominant leg after baseline measurements. The dominant leg was determined by asking the participants which leg they use to land when blocking or spiking the volleyball.

#### 2.4.1 Tissue flossing protocol

**FIGURE 2:**
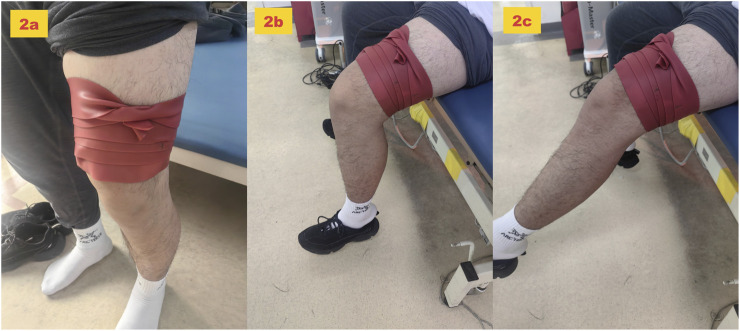
Tissue flossing application protocol. **(a)** Wrapping method. **(b)** felxion position. **(c)** extension position.

A natural rubber floss band (5.1 cm × 3.5 m, Sanctband COMPREFloss Blueberry; Sanct Japan Co., Ltd) was used. With the participant standing in slight knee flexion, the band was wrapped, starting from the lateral femoral epicondyle of the dominant leg and extending proximally to 5 cm above the lateral femoral condyle. Wrapping proceeded from the distal to proximal position with 50% band tension and 50% overlap between the layers to generate pressure (Figure 2a). Participants were then instructed to sit on a chair and perform slow, small-range knee flexion–extension movements (metronome-guided, 0.5 Hz, 30 cycles/minute, one cycle = one full knee flexion–extension) to facilitate hamstring gliding under the band (Wu et al., 2022). The band was removed immediately after 2 min (flexion position, Figure 2b extension position, Figure 2c).

#### 2.4.2 Dynamic stretching protocol

The participants stood upright with their feet parallel and facing a platform approximately level with their anterior superior iliac spine, placing their dominant leg on the platform (original standing position). The participants were then instructed to contract their hip flexors intentionally once every 2 s, causing their hip joints to flex while keeping their knees extended so that their dominant leg swung up to the anterior aspect of their body and their hamstrings were stretched (metronome-guided, 30 cycles/minute, 0.5 Hz) (Iwata et al., 2019; Kaneda et al., 2020). The participants stopped the stretching movement after 2 min.

#### 2.4.3 Placebo group protocol

Participants performed only 2 min of active knee flexion–extension movements of the dominant leg, which were identical in method and cadence (metronome-guided, 0.5 Hz, 30 cycles/minute) to the TF protocol but without the floss band (Kaneda et al., 2020).

### 2.5 Measurement

For all participants at the three time points, hamstring stiffness was measured first, followed by hamstring flexibility. The entire sequence of measurements (both stiffness and flexibility assessments) was completed in approximately 8 min per participant at each time point.

#### 2.5.1 Assessment of hamstring flexibility

The primary outcome of hamstring flexibility was the passive knee extension (KE) test (Reurink et al., 2013). The participant was placed in the supine position on an examination bed, with both the hip and knee positioned in 90° of flexion, and the non-tested leg was stabilized by a researcher to avoid compensatory movements. Hip flexion was maintained by another researcher holding the distal thigh. The participant was stabilized and instructed to remain relaxed and avoid any voluntary muscle contractions. The researcher extended the knee until maximal stretch within the tolerance of the hamstrings, with the ipsilateral hip remaining in 90° of flexion. The goniometer axis was aligned with the lateral femoral condyle. The remaining knee flexion angle represented hamstring tightness (Gajdosik and Lusin, 1983). This procedure was repeated three times, and the average of all three measures was recorded.

Secondarily, we also measured the passive straight leg raise (SLR) test, which served as an indicator of hamstring flexibility (López et al., 1994). The participant was placed in the supine position on an examination bed with the legs straight, and a researcher stabilized the non-tested leg to avoid compensatory movements. The participant was stabilized and instructed to remain relaxed and avoid any voluntary muscle contractions. The axis of a goniometer was aligned with the greater trochanter projection. The researcher raised the participant’s leg, with the knee passively extended, until the height where the participant felt a strong but tolerable stretch (Brusco et al., 2018). The other goniometer arm was aligned with the fibular head and lateral malleolus, and the hip flexion angle was recorded. This procedure was repeated three times, and the average of all three measures was recorded.

The forward flexion distance (FFD) test was used as an indicator of hamstring flexibility. The participants stood barefoot on a measurement box. They performed maximal trunk forward flexion with the knees and arms extended, palms parallel, and fingers stretched (López and Girela, 2003). The distance (cm) from the distal fingertips to a millimeter ruler placed vertically beside the box was measured using a portable tape measure. This test has been reported to have good validity and reliability (López and Girela, 2003). This procedure was repeated three times, and the average of all three measures was recorded.

#### 2.5.2 Assessment of hamstring stiffness

**FIGURE 3:**
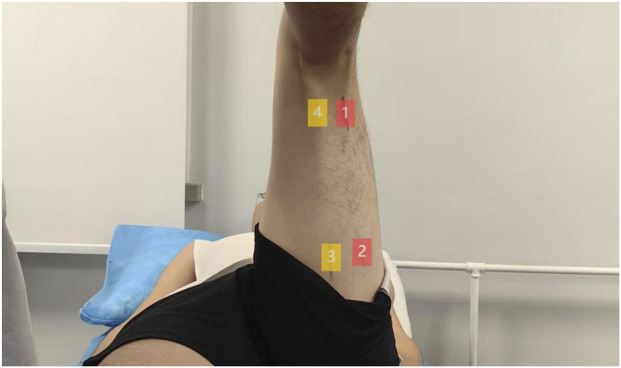
Schematic diagram of the measurement site. 1 represents the distal end of BFlh, 2 represents the proximal end of BFlh, 3 represents the proximal end of ST, and 4 represents the distal end of ST.

**FIGURE 4:**
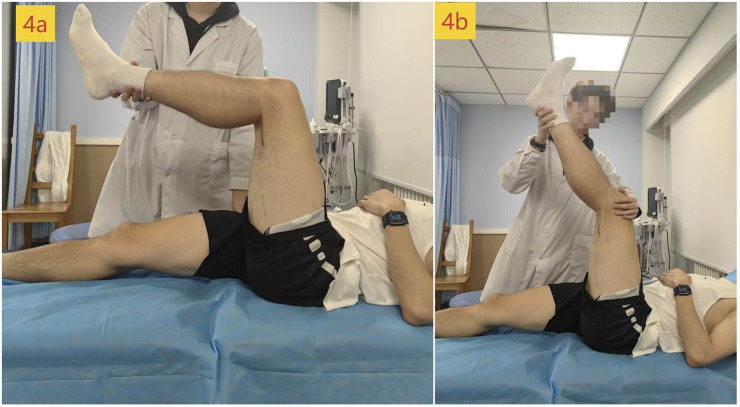
Position of measurement: **(a)** original standing position; **(b)** passive maximum stretch position.

Hamstring stiffness was assessed as the primary outcome using two-dimensional ultrasound SWE. Young’s modulus (kPa) of the distal (75% of the muscle belly length) and proximal (25% of the muscle belly length) regions of the biceps femoris long head (BFlh) and semitendinosus muscle (ST) of the dominant leg was measured in a passive maximum stretch position. The body marking for BFlh and ST was determined using conventional B-mode ultrasound in a prone position to identify the myotendinous junction. Distances were measured with a tape measure to locate the distal (75%) and proximal (25%) points along the muscle belly (Figure 3) (Miyamoto et al., 2020). During SWE measurement, participants were in the supine position. The pelvis was secured to the plinth with a non-elastic strap. An experimenter first passively positioned the dominant hip and knee at 90° flexion (the original standing position, Figure 4a). The knee was then slowly and passively extended by another experimenter until a predetermined endpoint was reached (Leung et al., 2025): the experimenter perceived a firm resistance to further knee extension (tautness) or (Dalton et al., 2015) the participant reported a strong but tolerable sensation of stretch (rated as 7/10 on a stretch intensity scale), whichever came first (the passive maximum stretch position, Figure 4b). The final knee extension angle achieved at this position was recorded for each participant and replicated at each subsequent assessment time point to ensure consistent muscle–tendon unit length during stiffness measurements.

**FIGURE 5:**
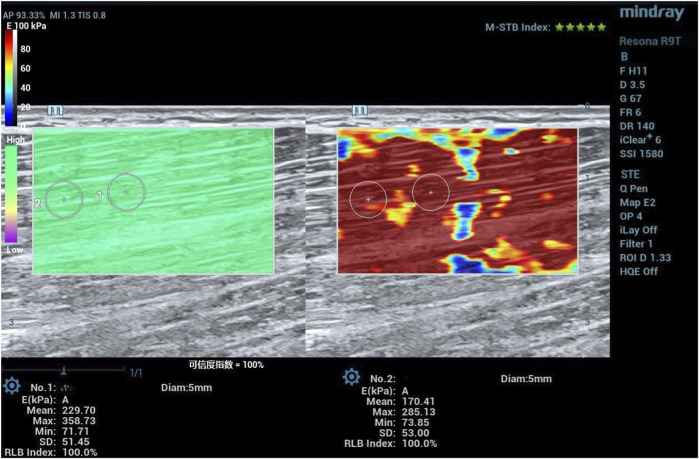
Measurement of shear-wave elastography (distal point of BFlh).

Young’s modulus was measured using a Mindray Resona R9T color Doppler ultrasound system (Shenzhen, China) equipped with an L15-3WU linear array probe in the SWE mode (musculoskeletal preset; probe frequency: 3 MHz–15 MHz; depth: 3 cm–5 cm). After positioning the probe, the system was stabilized for 5 s–8 s. An SWE image was considered stable and suitable for analysis when the motion stability index was ≥4, and the reliability index was ≥90% (see Figure 5 for an example SWE image). The image was frozen, and a quantitative analysis sampling box was manually placed to maximize muscle inclusion while avoiding myofascial junctions. A circular region of interest (ROI) with a 5-mm diameter was manually drawn within the homogeneous areas of the color-coded elastogram. The system automatically calculated the mean Young’s modulus and standard deviation (SD) within the ROI. Values with large deviations due to excessive probe pressure or muscle contraction were discarded (Miyamoto et al., 2020). Two measurements were taken per site, and the value with the lower SD was recorded as the result. All SWE measurements and ROI analyses were performed by an experimenter who received SWE operation training and had 2 years of experience in using ultrasound.

### 2.6 Sample size estimation and justification

Given the lack of established reference values for TF interventions in hamstring stiffness, we adopted a conservative approach for sample size estimation. Based on an *a priori* power analysis (G*Power 3.1) assuming a medium effect size (f = 0.50), with within–between interaction = 0.70, α = 0.05, power = 0.80, three repeated measurements, and an average correlation of 0.60 between measures, the calculated minimum sample size was 12 participants. To enhance statistical power and account for potential attrition, we ultimately enrolled 37 participants in this study.

### 2.7 Statistical analysis

Management and statistical analyses were performed with SPSS Statistics 26.0 software for Windows (SPSS Inc., Chicago, IL). The significance level was set at *p* < 0.05. Descriptive statistics (i.e., mean and SD) were used to summarize the demographic characteristics of the participants. Shapiro–Wilk tests were used to examine whether the data were normally distributed. Data that were normally distributed were described using “mean ± SD,” and those not normally distributed were described using “median (interquartile range)” (M (P25, P75)). The primary statistical analysis was designed to examine the effects of group (TF, DS, and placebo) and time (baseline, 0 min, and 30 min) on the outcome measures. Given the study’s mixed-factorial design (a between-subjects factor “group” and a within-subjects factor “time”), a two-way mixed-model ANOVA (also known as split-plot ANOVA) was initially planned. The Shapiro–Wilk test confirmed that all outcome variables significantly deviated from a normal distribution (*p* < 0.05). Since this violation precludes the use of parametric ANOVAs, we used generalized estimating equations (GEEs), which are a robust method for analyzing longitudinal data with non-normal distributions and do not require the strict assumptions of parametric tests. The GEE model included group, time, and their interaction term (group × time) as factors. When significant interactions were detected, *post hoc* pairwise comparisons with Bonferroni adjustment were conducted to examine between-group and within-group differences. For outcomes that did not demonstrate significant group × time interactions, a complementary analytical approach was used to examine potential treatment effects from different perspectives: within-group comparisons were performed by calculating the percentage change from baseline at each post-intervention time point using the formula: [(value at time point − baseline value)/baseline value] × 100%. These percentage change scores were then compared with groups using the Kruskal–Wallis (KW) test to examine whether the magnitude of improvement differed among the interventions. When significant overall differences were found, *post hoc* Dunn’s tests were applied to identify specific group differences. Between-group comparisons were conducted separately for each intervention group using the Friedman test to evaluate temporal effects across the three assessment points (baseline, 0 min, and 30 min). For outcomes with significant Friedman test results, *post hoc* analysis with Dunn’s test and Bonferroni adjustment was performed to identify specific pairwise differences between the time points. The 95% CIs and effect sizes for all variables were calculated. The reliability of the hamstring stiffness measurements was demonstrated by a single-rating, absolute-agreement, and two-way mixed-effects intra-class correlation coefficient (ICC) model. The strength of agreement was assessed using the ICC and has been previously described as follows: values less than 0.5 reflect poor reliability, between 0.5 and 0.75 reflect moderate reliability, between 0.75 and 0.9 reflect good reliability, and greater than 0.90 reflect excellent reliability (Koo and Li, 2016).

## 3 Results

All participants completed all treatments and assessments, and their data were included in the analysis. Demographic characteristics showed no significant differences between groups (*p* > 0.078, Table 1). Two male participants in the DS group experienced spasms of the hamstrings on the intervention side from 0 min to 30 min after the intervention, while the other participants did not have any adverse reactions during the experiment. The pre- and post-intervention values are shown in Table 2. Statistical analysis confirmed that all outcome measures (stiffness, KE, SLR, and FFD) did not follow normal distributions, as verified by Shapiro–Wilk tests (*p* < 0.05). Consequently, the GEE models and KW test models were utilized for comparative analysis. Baseline measurements demonstrated comparable pre-intervention status among the groups across all outcomes (*p* > 0.356, Table 3).

**TABLE 1:** Participant demographic characteristics.

Outcome	Total (n = 37)	TF group (n = 13)	DS group (n = 11)	Placebo group (n = 13)	*p*-value
Age^a^ (years)	24.0 (20.0, 29.0)	26.0 (21.0, 32.0)	20.0 (20.0, 29.0)	24.0 (22.5, 30.0)	0.078^b^
Sex (n)	M: 23/F: 14	M: 7/F: 6	M: 8/F: 3	M: 8/F: 5	
Height (m)	1.754 ± 0.105	1.763 ± 0.143	1.765 ± 0.074	1.735 ± 0.087	0.739
Weight (kg)	72.57 ± 16.96	73.11 ± 21.85	74.22 ± 16.66	70.62 ± 12.59	0.872
BMI (kg/m^2^)	23.30 ± 3.40	22.99 ± 3.56	23.60 ± 3.99	23.35 ± 2.92	0.913

M, male; F, female.

^a^ indicates that the outcome is described using the median (interquartile range).

^b^ indicates that the *p*-value was obtained through the KW test; others were obtained through one-way ANOVA.

**TABLE 2:** Pre- and post-intervention values.

Outcome	TF group (n = 13)			DS group (n = 11)			Placebo group (n = 13)		
	Pre	0 min	30 min	Pre	0 min	30 min	Pre	0 min	30 min
Stiffness (Kpa)									
BFlh (d)	71.14 (45.68, 124.21)	46.57 (33.61, 69.36)	40.66 (26.96, 58.62)	62.14 (56.92, 102.76)	55.45 (40.12, 86.81)	72.71 (66.15,119.05)	52.45 (40.97, 96.74)	60.08 (53.18, 87.62)	70.09 (61.33, 84.66)
BFlh (p)	50.74 (31.52, 76.45)	31.54 (24.76, 40.51)	25.93 (24.46, 32.83)	51.13 (26.36, 75.83)	34.86 (21.21, 77.84)	56.62 (39.07, 99.33)	40.00 (23.91, 77.40)	28.29 (20.08, 62.77)	43.73 (27.23, 73.65)
ST (d)	65.01 (45.54, 81.96)	43.80 (32.23, 56.43)	37.13 (29.96, 48.81)	100.06 (48.09, 120.77)	64.27 (38.58, 81.56)	76.68 (41.41,120.36)	72.04 (49.62, 102.31)	82.25 (68.97, 111.26)	80.66 (56.81, 111.42)
ST (p)	51.17 (36.88, 90.62)	35.86 (26.09, 61.49)	30.99 (23.03, 52.14)	94.83 (48.01, 114.04)	72.99 (38.52, 81.37)	77.87 (38.20,141.65)	41.34 (34.25, 69.88)	48.40 (40.23, 81.47)	45.26 (35.42, 77.63)
SLR (°)	79.0 (70.0, 87.5)	81.0 (79.0, 94.5)	87.0 (75.5, 96.0)	80.0 (65.0, 90.0)	90.0 (80.0, 101.0)	84.0 (70.0, 95.0)	75.0 (67.5, 80.0)	78.0 (69.0, 80.5)	75.0 (70.0, 86.5)
KE (°)	25.0 (14.0, 40.0)	15.0 (14.5, 27.5)	21.0 (14.5, 29.5)	30.0 (20.0, 46.0)	23.0 (12.0, 29.0)	31.0 (15.0, 35.0)	25.0 (19.0, 35.0)	25.0 (22.5, 33.0)	32.0 (18.5, 36.5)
FFD (cm)	11.00 (0.25, 15.50)	13.00 (14.50, 27.50)	11.50 (1.00, 15.00)	12.00 (0, 14.00)	12.00 (2.00, 16.00)	13.00 (−0.50, 17.00)	6.00 (−12.50, 10.00)	7.50 (−9.75, 10.00)	7.00 (−6.75, 9.75)

TF, tissue flossing; DS, dynamic stretching; BFlh, biceps femoris long head; ST, semitendinosus; d, distal; p, proximal; SLR, passive straight leg raise; KE, passive knee extension; FFD, forward flexion distance; Pre, baseline; 0 min, post-intervention 0 min; 30 min, post-intervention 30 min. Values are expressed as the median (interquartile range).

**TABLE 3:** Intergroup comparison of the test indicators.

Outcome/time point		Between-intervention differences (95% simultaneous CI)						Interaction effect	
		TF vs. DS	ES	TF vs. placebo	ES	DS vs. placebo	ES	F-value	*p*-value
Stiffness (Kpa)									
BFlh (d)	Pre	2.50 (−49.98, 54.99)	0.06	14.41 (−35.98, 64.81)	0.34	11.91 (−45.90, 36.60)	0.46	36.165	<0.001
	0 min	−9.49 (−45.66, 26.67)	0.33	−20.01 (-65.12, 25.10)	0.53	−10.51 (−56.42, 35.40)	0.28		
	30 min	−49.98 (−100.79, 0.82)	0.87	−38.64 (−72.72, -4.55)*	1.36	11.35 (−45.40, 68.09)	0.26		
BFlh (p)	Pre	−10.82 (−60.05, 38.41)	0.29	3.45 (−30.25,37.14)	0.12	14.27 (−38.41, 66.94)	0.35	43.546	<0.001
	0 min	−13.96 (−13.99, 41.91)	0.76	−9.43 (−37.65, 18.79)	0.40	4.53 (−33.72, 42.78)	0.15		
	30 min	−44.70 (−89.79, 0.39)	1.35	−26.92 (−61.13, 7.28)	0.95	17.78 (−38.37, 73.92)	0.52		
ST (d)	Pre	−23.54 (−62.69, 15.60)	0.79	−11.00 (−42.90, 20.90)	0.42	12.54 (−31.87, 56.96)	0.36	58.868	<0.001
	0 min	−20.35 (−45.87, 5.16)	1.04	−45.71 (−71.90, -19.52)*	2.10	−25.35 (−58.33, 7.62)	0.95		
	30 min	−43.40 (−80.65, -6.16)*	1.56	−45.03 (−76.35, -13.71)*	1.73	−1.63 (−48.43, 45.18)	0.04		
ST (p)	Pre	−25.14 (−75.37, 25.08)	0.65	7.72 (−27.48, 42.91)	0.26	32.86 (−15.86, 81.58)	0.88	33.343	<0.001
	0 min	−17.97 (−52.92, 16.99)	0.66	−17.24 (−50.62, 16.15)	0.89	0.73 (−37.56, 39.01)	0.02		
	30 min	−51.13 (−101.20, -1.05)^*^	1.35	−18.91 (−52.05, 14.24)	0.69	32.22 (−21.18, 85.62)	0.78		
SLR (°)	Pre	0.36 (−18.65, 19.36)	0.02	6.23 (−12.48, 24.94)	0.40	5.87 (−11.72, 23.47)	0.42	3.830	0.430
	0 min	−1.95 (−21.26, 17.36)	0.13	8.77 (−6.62, 24.16)	0.69	10.72 (−7.26, 28.70)	0.77		
	30 min	5.10 (−12.82, 23.01)	0.35	10.31 (−8.35, 28.97)	0.67	5.21 (−11.04, 21.46)	0.40		
KE (°)	Pre	−3.58 (−22.97, 15.81)	0.23	0 (−16.10, 16.10)	0	3.58 (−13.01, 20.17)	0.28	7.179	0.127
	0 min	−2.61 (−17.74, 12.52)	0.22	−7.46 (−19.53, 4.60)	0.74	−4.85 (−17.72, 8.01)	0.49		
	30 min	−4.92 (−19.88, 10.05)	0.42	−6.54 (−19.75, 6.67)	0.41	−1.62 (−16.82, 13.58)	0.04		
FFD (cm)	Pre	2.52 (−12.02, 17.06)	0.22	7.46 (−5.42, 20.34)	0.70	4.94 (−9.44, 19.33)	0.44	3.634	0.458
	0 min	2.58 (−11.21, 16.38)	0.23	8.65 (−4.57, 21.88)	0.79	6.07 (−7.78, 19.92)	0.55		
	30 min	1.25 (−11.61, 14.10)	0.12	7.27 (−5.36, 19.90)	0.69	6.02 (−6.63, 18.68)	0.59		

TF, tissue flossing; DS, dynamic stretching; BFlh, biceps femoris long head; ST, semitendinosus; d, distal; p, proximal; SLR, passive straight leg raise; KE, passive knee extension; FFD, forward flexion distance; ES, effect size (Cohen’s d); Pre, baseline; 0 min, post-intervention 0 min; 30 min, post-intervention 30 min; * indicates a significant within-group difference at the same time point by the generalized estimating equation.

### 3.1 Effects of TF and DS on hamstring stiffness

The GEE models demonstrated a significant interaction between the group and time at the distal and proximal regions of BFlh and ST (F ≥ 33.343, *p* < 0.001). *Post hoc* analyses showed that the stiffness of the TF group was significantly lower than that of the DS group at the distal and proximal regions of ST at post-intervention 30 min [ST(d): mean difference = −43.40 Kpa, 95% CI: (−80.65, −6.16), *p* = 0.007, Cohen’s d = 1.56/ST(p): mean difference = −51.13 Kpa, 95% CI: (−101.20, −1.05), *p* = 0.040, Cohen’s d = 1.35]. Meanwhile, the *post hoc* analyses also showed that the stiffness of the TF group was significantly lower than that of the placebo group at the distal region of BFlh at post-intervention 30 min [mean difference = −38.64 Kpa, 95% CI: (−72.72, −4.55), *p* = 0.010, Cohen’s d = 1.36] and at the distal region of ST at post-intervention 0 min [mean difference = −45.71 Kpa, 95% CI: (−71.90, −19.52), *p* < 0.001, Cohen’s d = 2.10]. However, there was no significant difference between the stiffness of the TF group and that of the DS or placebo groups at other test sites of BFlh or ST post-intervention (*p* ≥ 0.055).

Intergroup analysis showed that the stiffness at all measurement regions in the TF group decreased significantly at both post-intervention 0 min and 30 min compared with that at baseline (*p* < 0.001), and even at the proximal region of BFlh, the stiffness at post-intervention 30 min also decreased significantly compared with that at post-intervention 0 min [mean difference = −5.43 Kpa, 95% CI: (−9.67, −1.19), *p* = 0.007]. However, the stiffness in the DS group only decreased significantly at post-intervention 0 min compared with that at baseline [p:ST(d) = 0.004/ST(p) = 0.007/BFlh(p) = 0.036] but returned to the pre-intervention level [ST(d): mean difference = −4.74 Kpa, 95% CI: (−25.74, 16.26), *p* = 1.000/ST(p): mean difference = 3.75 Kpa, 95% CI: (−22.57, 30.07), *p* = 1.000/BFlh(d): mean difference = 12.33 Kpa, 95% CI: (−2.15, 26.83), *p* = 0.125] or increased significantly at post-intervention 30 min [BFlh(p): mean difference = 7.04 Kpa, 95% CI: (0.35, 13.74), *p* = 0.035].

The ICC for the hamstring stiffness SWE measurements was 0.86 (*p* < 0.001), indicating good consistency.

### 3.2 Effects of TF and DS on hamstring flexibility

**FIGURE 6:**
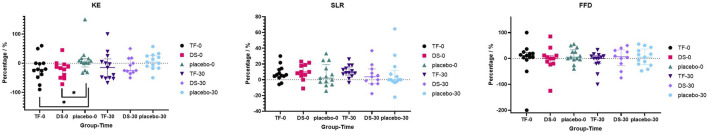
Scatter plots of the percentage differences from baseline in KE, SLR, and FFD at post-intervention 0 and 30 min in each group.

The GEE models demonstrated no significant group-by-time interaction effect of KE (F = 7.719, *p* = 0.127), SLR (F = 3.830, *p* = 0.430), and FFD (F = 3.634, *p* = 0.458). Therefore, the KW test demonstrated that only the percentage changes in KE from baseline to post-intervention 0 min (H = 6.708, *p* = 0.035) in the TF group (median change: −21.1%, IQR: −42.5% to 0.0%, *p* = 0.022) and the DS group (median change: −16.7%, IQR: −50.0% to −4.4%, *p* = 0.032) significantly decreased compared to those in the placebo group (median change: +8.7%, IQR: −12.5 to +18.3%). The other percentage changes in KE, SLR, or FFD from baseline to post-intervention 0 or 30 min did not differ significantly among the three groups [KE (30 min): H = 3.181, *p* = 0.204/SLR (0 min): H = 1.657, *p* = 0.437/SLR (30 min): H = 3.863, *p* = 0.145/FFD (0 min): H = 0.527, *p* = 0.768/FFD (30 min): H = 0.735, *p* = 0.693]. The percentage changes in SLR, FFD, and KE are shown in Figure 6.

Intergroup analysis (Friedman test) showed significant temporal effects of SLR and FFD (SLR: X^2^ = 14.596, *p* = 0.001/FFD: X^2^ = 12.160, *p* = 0.002) but a non-significant temporal effect of KE (X^2^ = 4.217, *p* = 0.121) in the TF group. *Post hoc* Dunn’s tests revealed that in the TF group, the value of FFD at post-intervention 0 min increased significantly compared with that at baseline (*p* = 0.001), and the values of SLR at post-intervention 0 and 30 min both increased significantly compared with that at baseline (0 min: *p* = 0.018/30 min: *p* = 0.001). The Friedman test also showed a significant temporal effect of KE (X^2^ = 8.390, *p* = 0.015) but a non-significant temporal effect of SLR (X^2^ = 4.874, *p* = 0.087) and FFD (X^2^ = 5.463, *p* = 0.065) in the DS group. *Post hoc* Dunn’s tests revealed that in the DS group, the value of KE at post-intervention 0 min decreased significantly compared with that at baseline (*p* = 0.017).

## 4 Discussion

While SWE has been increasingly utilized to investigate the effects of various stretching modalities on muscle stiffness (Hirata and Akagi, 2024; Železnik et al., 2024), its application specifically to evaluate TF, particularly in direct comparison with DS, on the hamstrings, remains limited. To our knowledge, this study is among the first to utilize SWE to directly and quantitatively compare the acute and sustained effects of TF and DS on hamstring muscle stiffness. In our study, TF was more persistently effective at improving hamstring stiffness than DS, but it did not show superior performance in hamstring flexibility. The therapeutic difference between TF and DS in stiffness was not manifested in flexibility, potentially demonstrating the principle of the different mechanisms of action of TF and DS on the hamstrings.

Although previous studies of TF have shown more improvement in hamstring stiffness, there are still many limitations (Kaneda et al., 2020; Wu et al., 2022). They all proposed that TF could improve hamstring stiffness immediately after intervention, which was assessed by an isokinetic dynamometer, but the continuous improvement effect of TF was never mentioned. Using the more precise SWE technique, our findings demonstrate that TF has a better improvement effect on stiffness (ST(d)) than DS at 30 min after intervention, suggesting that TF may continuously release the fascia tissue of hamstrings. Although both warm-up methods demonstrated comparable stiffness decrease, TF intervention appears particularly advantageous for continuously improving hamstring stiffness after intervention. This was further confirmed by the within-group changes in the TF and DS groups. After TF intervention, the hamstring stiffness showed a continuous downward trend over time, while after DS intervention, the hamstring stiffness decreased greatly but returned to the pre-intervention level at 30 min after intervention. As the game progresses, athletes experience fatigue, so the incidence of HS in the second half of the game is significantly higher than that in the first half (Yagiz et al., 2022; Huygaerts et al., 2020). These findings suggest that TF warrants consideration as a warm-up method for athletes, and its potential to sustain reduced stiffness in a rested state merits investigation into how it interacts with subsequent athletic performance and fatigue.

The superior sustained reduction in hamstring stiffness observed following TF, as opposed to that following DS, may be attributed to its distinct mechanisms of action. While DS primarily influences neuromuscular adaptability and viscoelastic properties through active movement (Vieira et al., 2021; Fukaya et al., 2025; Ingram et al., 2025), TF is postulated to exert its effects through multiple mechanisms. The compressive force applied by the floss band may first induce a brief period of ischemia, followed by reactive hyperemia upon its release, potentially improving local microcirculation and clearing metabolic byproducts (Paravlic et al., 2022; Schneider et al., 2025). Furthermore, the mechanical shear and gliding forces generated under the band during movement may facilitate the release of fascial adhesions and alter the thixotropic properties of the myofascial tissue (Maemichi et al., 2025; Cheatham and Baker, 2024; Konrad et al., 2021). Thixotropy refers to the property of certain gels or fluids to become less viscous under mechanical stress (e.g., shear from movement) and return to a more viscous state at rest. TF may ‘reset’ the thixotropic state of the musculofascial unit, thus reducing passive stiffness for a prolonged period (Maemichi et al., 2025; Konrad et al., 2021). This alteration in the fundamental biophysical properties of the tissue, as opposed to a transient neural adaptation, provides a plausible explanation for the sustained (30-min) reduction in stiffness observed in the TF group. This mechanistic focus on the fascial and fluid properties of the tissue, rather than solely on neuromuscular length tolerance, could explain why a significant reduction in stiffness (as measured by SWE) did not fully translate to commensurate improvements in traditional flexibility measures such as KE or SLR, which are influenced by both mechanical and neural factors.

Contrary to the findings of Kaneda et al. (2020), who reported superior improvements in passive KE following TF compared to DS, our study observed no significant between-group differences in flexibility (KE, SLR, and FFD). This discrepancy could be attributed to differences in the flexibility assessment protocols (e.g., FFD vs. passive KE/SLR) or the characteristics of the study population (e.g., trained individuals vs. our cohort of light volleyball enthusiasts). The KE and SLR tests, while standard, measure a composite of musculoskeletal flexibility and neural tolerance to stretch (Ingram et al., 2025), which might be less sensitive to the specific fascial and fluid mechanical changes induced by TF than SWE-derived stiffness measures.

Several limitations should be considered when interpreting our findings. First, the female sample size in this study was relatively small. Although the participants in each group had met the calculated *a priori* sample size, the reduced statistical power after subgroup analysis was a concern; therefore, our study did not perform gender-based subgroup analyses. This might be the reason why we chose the GEE model for analysis, which reduced the efficacy of the study itself. Second, the optimal TF dosage and frequency remain undetermined, requiring future dose–response studies. Finally, this study focused on the changes in tissue stiffness at the distal and proximal ends of BFlh and ST at 30 min after intervention. There was a lack of records of tissue stiffness in other parts of the hamstring muscle and for longer observation periods. In future studies, changes in tissue stiffness in other parts of the hamstring muscles can be examined, and the observation period can be appropriately extended.

## 5 Conclusions

This study provides evidence that compared with DS, TF can continuously improve hamstring stiffness in LVB enthusiasts, suggesting its potential as a warm-up protocol. However, no significant flexibility differences between TF and DS were observed, possibly due to the small female sample size. These findings should be interpreted cautiously until confirmed by larger, methodologically robust trials with longer-term assessments.

## Data Availability

The raw data supporting the conclusions of this article will be made available by the authors, without undue reservation.
